# CXCL12-CXCR4 pathway activates brown adipocytes and induces insulin resistance in CXCR4-deficient mice under high-fat diet

**DOI:** 10.1038/s41598-019-42127-8

**Published:** 2019-04-16

**Authors:** Kenichi Kurita, Ko Ishikawa, Kenji Takeda, Masanori Fujimoto, Hiraku Ono, Jin Kumagai, Hiromi Inoue, Hidetaka Yokoh, Koutaro Yokote

**Affiliations:** 1 Department of Endocrinology, Hematology and Gerontology, Chiba University Graduate School of Medicine, 1-8-1 Inohana, Chuo-ku, Chiba, 260-8670 Japan; 2 Division of Diabetes, Metabolism and Endocrinology, Chiba University Hospital, 1-8-1 Inohana, Chuo-ku, Chiba, 260-8670 Japan

## Abstract

Brown adipose tissue (BAT) plays a role in energy expenditure and is involved in nutrient metabolism. C-X-C chemokine ligand 12 (CXCL12)-CXCR4 pathway regulates the immune, nervous, and cardiovascular systems and affects the adipose tissue. Here, we investigated the role of this pathway as an activator of BAT. Uncoupling protein 1 mRNA and protein levels and oxygen consumption increased in the brown adipocytes treated with 100 nM CXCL12 peptide. CXCL12-mediated upregulation in P38 and extracellular signal-regulated kinase (ERK) levels was reduced by each inhibitor. Thus, the CXCL12-CXCR4 pathway activated the brown adipocytes through P38 and ERK that acted downstream of this pathway. Mice with CXCR4 defects only in the brown adipocytes were generated and fed with high-fat diet (HFD). Body weight and blood glucose after glucose injection increased in these mice. Long-term exposure to HFD deteriorated blood glucose level after glucose injection. Insulin sensitivity was exacerbated in the knockout mice fed with HFD. Serum lipid parameters and CXCL12 level in knockout mice were similar to those in control mice. These results suggest that the CXCL12-CXCR4 pathway induces brown adipocyte activity and affects nutrient metabolism under HFD load.

## Introduction

Brown adipose tissue (BAT) dissipates energy in the form of heat to maintain body temperature and energy balance^1^. Glucose or fatty acid is oxidised in brown adipocytes for thermogenesis in response to cold or high-caloric diet. Hence, brown adipocyte activity contributes to basic metabolism via energy expenditure through glucose or fatty acid consumption^1–3^. Noradrenaline is a canonical activating factor of brown adipocytes that is released from the sympathetic nerve terminal as a neurotransmitter in response to cold. Many new activating factors have been reported in brown adipocytes, such as bile acids, natriuretic peptides, bone morphogenetic protein (BMP)-8b, irisin, and fibroblast growth factor (FGF)-21^4^. Mammals with highly active brown adipocytes develop resistance to body weight gain, hyperglycaemia, and hyperlipidaemia, or exhibit resistance to metabolic syndrome. Thus, the activation of brown adipocytes may serve as one of the strategies to combat obesity, diabetes mellitus, and hyperlipidaemia^5^.

C-X-C chemokine ligand 12 (CXCL12) is one of the chemokines also known as stromal-derived factor (SDF)-1. CXCL12 is indispensable, owing to the high homology in the sequence of this protein from several species. CXCR4, a receptor of CXCL12, is a type of G coupling protein receptor. The CXCL12-CXCR4 pathway plays a pivotal role in cell migration, haematopoiesis, cardiovascular development, lymphoid organ morphogenesis, and neural differentiation during embryonic stage^6,7^. Furthermore, this pathway is involved in various neoplasms such as multiple myeloma, acute myeloid leukaemia, diffuse large B cell lymphoma, adrenocortical cancer, and small lung cancer^8^.

The CXCL12-CXCR4 pathway was recently reported to affect energy metabolism. CXCL12 encourages β cell survival in islets^9^. Serum levels of CXCL12 were higher in patients with type 2 diabetes than in healthy subjects^10^. This pathway deteriorated non-alcoholic steatohepatitis (NASH) through CD4^+^ T cell recruitment^11^. The CXCR4 expresses in white adipocytes^12^. CXCL12 is secreted from mature white adipocytes and induces macrophage infiltration in the white adipose tissue (WAT), owing to adipocyte accumulation^13^. The infiltrated macrophages secrete proinflammatory cytokines and cause insulin resistance. Furthermore, CXCL12 from white adipocytes directly deteriorate insulin sensitivity of the parent cells^14^.

A knockout mouse with CXCR4 ablation created using the Cre-lox P system with fatty acid-binding protein 4 (FABP4) Cre mouse was prone to obesity and hyperglycaemia caused by macrophage infiltration into the WAT and hypoactivity of brown adipocytes^15^. However, the effect of CXCL12 on brown adipocytes has not been clarified. Whether the CXCL12-CXCR4 pathway influences only the brown adipocytes is not well documented, as FABP4 as a Cre promoter is generally expressed in both white and brown adipocytes, with minor expression level also detected in macrophages.

To evaluate the effect of the CXCL12-CXCR4 pathway on brown adipocytes, we determined the brown adipocyte activation by CXCL12 and metabolic state in mice with CXCR4 defects only in brown adipocytes.

## Results

### Property of the immortalised brown adipocytes generated from the BAT of C57/B6 mice

**Figure 1:**
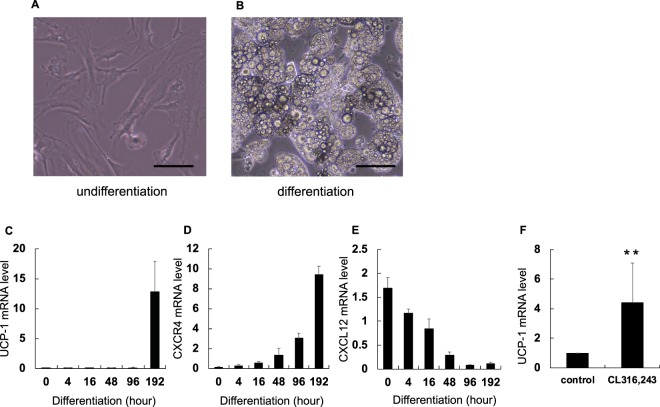
Immortalised brown adipocytes (CB-1 cells) was established as a culture cell line. Immortalised brown adipocytes were generated from primary brown adipocytes using SV40 Lentivirus. Induction of differentiation was performed with differentiation medium. RNAs were extracted as mentioned in the method part. Expression of mRNA extracted from CB-1 cells was examined by qRT-PCR and normalised to *36B4* gene. Values expressed as mean ± SD. (**A**,**B**) Picture of CB-1 cells at day 1 and day 2 after induction of differentiation. Scale bar represents 100 μm. (**C**–**E**) Levels of *UCP-1*, *CXCR4*, and *CXCL12* mRNA with differentiation of CB-1 cells, respectively. (**F**) Levels of *UCP-1* mRNA expression in well-differentiated CB-1 cells. CL316,243 was an agonist of β3 adrenergic receptors as the neurotransmitter from the terminus of the sympathetic nerve. **P < 0.01 (n = 5). The parameter was evaluated with the two-tailed unpaired Student’s *t*-test.

Immortalised brown adipocytes (CB-1 cells) obtained using SV40 Lenti virus had the ability to store multi-ocular lipid droplets (Fig. 1A,B). Uncoupling protein (UCP)-1 is a crucial molecule involved in brown adipocyte activity and acts as a surrogate marker of brown adipocyte activity. *UCP-1* and *CXCR4* mRNA expression levels increased after the induction of adipocyte differentiation (Fig. 1C,D).

Figure 1E shows the decrease in *CXCL12* mRNA expression upon differentiation. *UCP-1* mRNA level was significantly elevated in the CB-1 cells treated with β3 adrenergic receptor (B3AD-R) agonist, CL316,243, as compared with the control cells (Fig. 1F). CL316,243 is one of the most intensive activators of brown adipocytes. Therefore, we used well-differentiated CB-1 cells in these experiments, owing to their characteristic similarity with brown adipocytes.

### Activation of brown adipocytes increases with CXCL12 expression in CB-1 cells

**Figure 2:**
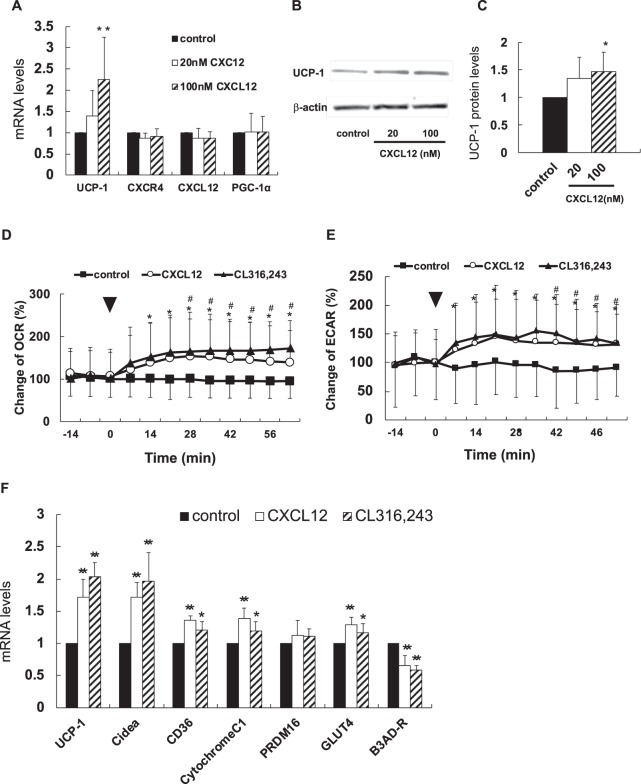
A CXCL12 promotes brown adipocyte activity. Mature CB-1 cells were treated with 100 nM of recombinant CXCL12 for 6 h. Bonferroni/Dunn test was used to compare the difference between the three groups. *P < 0.05, **P < 0.01 versus control. Values are shown as mean ± SD. (**A**) Changes in the expression of each gene when recombinant CXCL12 was added to brown adipocytes. (n = 3–4). Black, open, and stripe bar are shown as control, 10 nM, 100 nM of CXCL12, respectively. (**B**,**C**) Changes in expression of UCP-1 protein in CB-1 cells treated with the CXCL12 (n = 5). Black, open, and stripe bar are shown as control, 10 nM, 100 nM of CXCL12, respectively. (**D**,**E**) Oxygen consumption rate (OCR) when recombinant CXCL 12 was added to brown adipocytes (n = 20). Extracellular Acidification Rate (EACR) in CB-1 cells with CXCL12 (n = 20). Black arrow shows the point of addition of CXCL12. Black square, open circle, and black triangle are shown as control, CXCL12, and CL316,243. *P < 0.05. CL316,243 versus control, ^#^P < 0.05 CXCL12 versus control. (**F**) Various mRNA expression levels in CB-1 cells treated with CXCL12 (n = 6). Black, open, and stripe bar are shown as control, CXCL12, and CL316,243.

The expression levels of UCP-1 gene and protein increased in the CB-1 cells treated with 100 nM of recombinant CXCL12 (Fig. 2A–C). Furthermore, oxygen consumption rate (OCR) and extracellular acidification rate (ECAR) significantly increased in the CB-1 cells treated with both CXCL12 and CX316,243 as compared with control cells (Fig. 2D,E). Thus, CXCL12 directly induced brown adipocytes through increased oxygen consumption in the mitochondria and enhanced glycolytic pathway.

Figure 2 shows the mRNA expression levels of some genes in the CB-1 cells stimulated with 100 nM of recombinant CXCL12. The expression levels of UCP-1, cell death activator Cidea, and B3AD-R as surrogate markers of thermogenesis increased. Elevation in the mRNA levels of cytochrome C1 suggests the activation of the mitochondrial respiratory chain by CXCL12. An increase in *CD36* and *GLUT4* mRNA levels indicates the availability of fatty acid or glucose uptake as a substrate for thermogenesis in brown adipocytes. The static expression of PR domain zinc finger protein 16 (*PRDM16*) suggests the lack of its association with the differentiation of brown or beige adipocytes.

### Cell signalling pathway stimulated by CXCL12 in CB-1 cells

**Figure 3:**
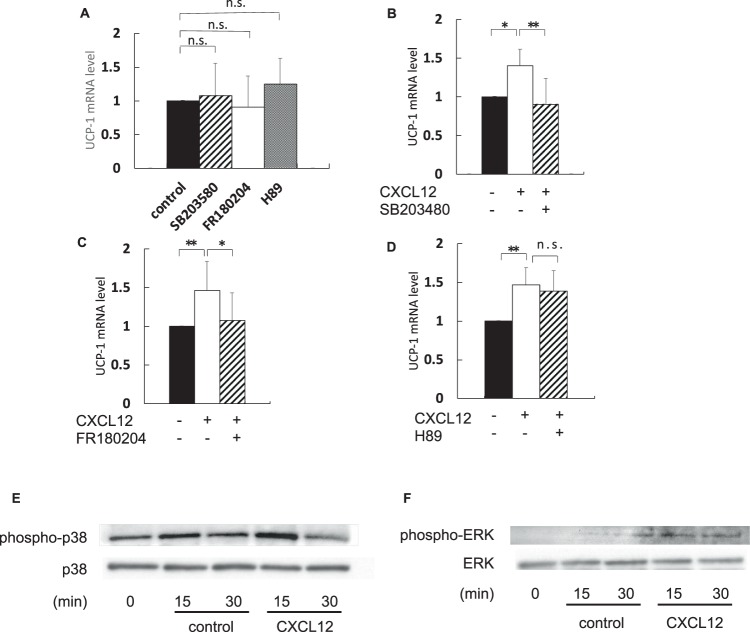
CXCL12-CXCR4 system works via p38 and ERK signalling. Well-differentiated CB-1 cells were added with 100 nM of CXCL12 for 2 h after preincubation with signal inhibitors at 37 °C for 30 min. RNAs were extracted as mentioned in the methods section. qRT-PCR was performed to estimate expression of *UCP-1* mRNA normalised to *36B4* gene. Bonferroni/Dunn test was used to compare the difference between the three groups. *P < 0.05, **P < 0.01 versus control. Values are shown as mean ± SD. Black, stripe, open, and dot bar are shown as control, SB203580, FR180204, and H89, respectively. n.s.: not a significant difference. SB203480; P38 inhibitor, FR180204; extracellular signal-regulated kinase (ERK) inhibitor, H89; protein kinase A (PKA) inhibitor. (**A**) *UCP-1* mRNA expression in CB-1 cells with each inhibitor alone (n = 6–9). (**B**–**D**) *UCP-1* mRNA expression in CB-1 cells treated with recombinant CXCL12 or/and each inhibitor (n = 6–9). (**E**) Protein expression of phosphorylated p38 and total p38 in CB-1 with CXCL12. (**F**) Protein expression of phosphorylated ERK and total ERK in CB-1 with CXCL12.

Figure 3A shows that the effect of each inhibitor was independent of *UCP-1* mRNA expression. The expression of *UCP-1* mRNA elevated by CXCL12 treatment decreased after treatment with a P38 inhibitor, SB203480 (Fig. 3B). Furthermore, *UCP-1* mRNA expression also decreased in the presence of an extracellular signal-regulated kinase (ERK) inhibitor, FR180204 (Fig. 3C). On the contrary, the increase in *UCP-1* mRNA level by CXCL12 treatment was unaffected by a protein kinase A (PKA) inhibitor, H89 (Fig. 3D). These results suggest that the CXCL12-CXCR4 pathway activated brown adipocytes via both P38 and ERK but not PKA.

Phosphorylated p38 increased at 15 minutes after adding CXCL 12 to CB-1 cells. On the other hand, phosphorylated ERK increased at 15 minutes and 30 minutes after addition of CXCL12 compared with control (Fig. 3E,F).

### Expression of mRNA in the BAT under some conditions

**Figure 4:**
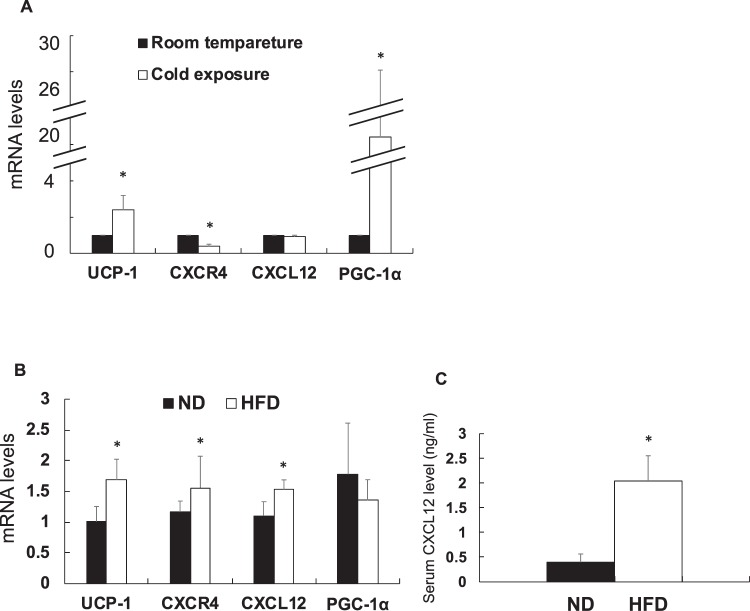
Expression of mRNA in brown adipose tissue under the high-fat diet loading or the cold condition C57/B6 mice were placed in the room at 4 °C or 28 °C for 4 h. On the other hand, C57/B6 mice were fed 60 kcal% of fat diet or 5 kcal% of fat diet. Various gene expression was examined using qRT-PCR and normalised to *36B4* gene. Values are shown as mean ± SD. Student’s *t*-test was used to compare the difference. *P < 0.05, **P < 0.01. (**A**) Expression of mRNA in the cold condition compared with room temperature (n = 4). Black and open bar represents room temperature and cold. (**B**) mRNA levels in the high-fat diet loading compared with normal diet (n = 9). ND: normal diet; FHD: high-fat diet. Black and open bar represents normal diet group and high-fat diet group. (**C**) Serum CXCL12 levels in ND group and HFD group (n = 9).

The mRNA levels of *UCP-1* and peroxisome proliferator-activated receptor gamma coactivator 1 alpha (*PGC-1α*) increased in the BAT of C57/B6 mice at 4 °C than at 28 °C (Fig. 4A). No difference was observed in the expression levels of *CXCR4* and *CXCL12* mRNAs at cold and room temperature (28 °C) conditions. Moreover, *UCP-1*, *CXCR4*, and *CXCL12* mRNA levels increased in the BAT of the mice fed with high-fat diet (HFD) as compared with those fed with normal diet (ND) (Fig. 4B). Serum CXCL12 concentration was higher in HFD group than in ND group (Fig. 4C).

### Generation of BAT-specific CXCR4-deficient mice

**Figure 5:**
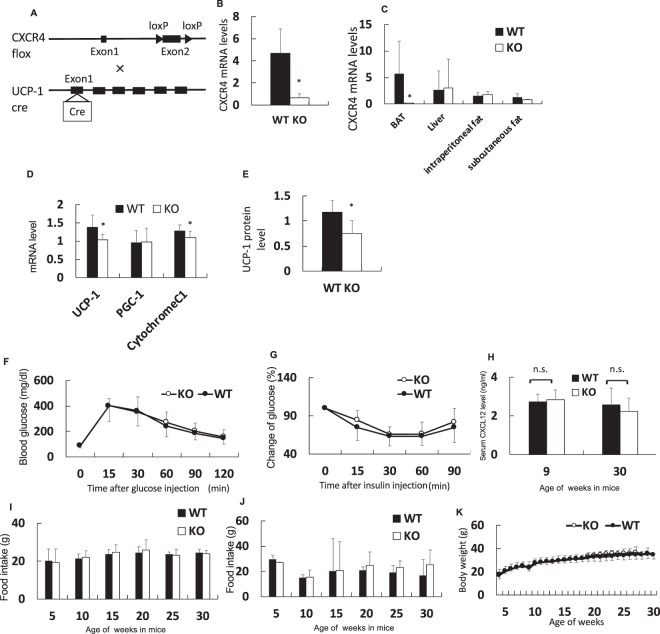
Brown adipocyte-specific CXCR4-deficient mice were generated. Expression of mRNA extracted from each tissue or primary brown adipocyte was evaluated using qRT-PCR and normalised to *36B4* gene. Serum CXCL12 concentration was measured using the Quantikine ELISA Mouse CXCL12/SDF-1α. Values are shown as mean ± SD. Student’s *t*-test was used to compare with a difference. *P < 0.05. n.s.: not a significant difference. Black and open bar or circle represent control and the knockout mice group, respectively. WT: wild-type, CXCR4^flox/−^mice; KO: knockout, CXCR4^flox/flox^mice (**A**) knockout mouse in using the Cre-loxP system. Exon 2 of CXCR4 was deleted in the BAT expressed *UCP-1* gene. (**B**) *CXCR4* mRNA expression in primary brown adipocyte from CXCR4^flox/flox^ compared with CXCR4^flox/−^ (n = 3). (**C**) Expression of *CXCR4* gene in the brown adipose tissue (BAT), Liver, Intraperitoneal white adipose tissue (WAT), and subcutaneous WAT (n = 7). (**D**) *UCP-1, PGC-1α, and Cytochrome C1* mRNA expression in the BAT of CXCR4^flox/−^ and CXCR4^flox/flox^ mice (n = 6). (**E**) UCP-1 protein level in the BAT of CXCR4^flox/−^ and CXCR4^flox/flox^ mice (n = 4–6). (**F**) Intraperitoneal glucose tolerance test in the mice with normal chow diet. (**G**) Insulin tolerance test in the mice with normal chow diet. (**H**) Serum CXCL12 concentration in mouse at nine weeks old (n = 6–5) and 30 weeks old (n = 9–7). (**I**) Change of food intake in normal chow diet. (**J**) Change of food intake in the high-fat chow diet. (**K**) Change of body weight in the mouse with normal chow diet.

HFD, but not cold circumstances, enhanced the expression of the CXCL12-CXCR4 pathway in brown adipocytes and resulted in systemic elevation in serum levels of CXCL12. Hence, we generated mice with CXCR4 ablation only in the brown adipocytes using Cre UCP-1 promoter, as CXCR4 deletion in the whole body is lethal owing to dysgenesis of the cardiovascular and nervous systems (Fig. 5A)^16,17^. *CXCR4* mRNA level decreased in the primary brown adipocytes prepared from the BAT of CXCR4-deficient mice than in those prepared from the BAT of C57/B6 mice (Fig. 5B). Therefore, brown adipocytes in the knockout mice expressed no CXCR4 gene and protein.

The expression levels of *CXCR4* showed no changes in other tissues such as the liver and intraperitoneal and subcutaneous WAT in the knockout mice (Fig. 5C). Figure 5D reveals the reduction *UCP-1* mRNA levels in the BAT of CXCR4-deficient mice compared with control mice. UCP-1 protein levels also decreased in the knockout mice (Fig. 5E). The level of UCP-1, a critical molecule of brown adipocyte activity, also reduced in the knockout mice. *PGC-1α* mRNA was no difference in BAT, however *Cytochrome C1* mRNA in BAT of knockout mouse was less than that of control (Fig. 5D). These results suggest a decrease in BAT function in the knockout mouse.

Glucose intolerance by intraperitoneal glucose tolerance test (IPGTT) and insulin tolerance test (ITT) was no significant difference between knockout mouse and control mouse with normal chow (Fig. 5F,G). No histological difference was observed in the BAT and WAT of both groups (Supplementary Fig. S1). Furthermore, no difference was observed in the serum concentration of CXCL12 at 9 or 30 weeks between two groups (Fig. 5H). Furthermore, we evaluated food intake and weight change in the normal chow diet, but there was no difference between wild type and knockout mice (Fig. 5I,K). There was also no difference between wild type and the knockout mice with high-fat chow diet (Fig. 5J).

UCP-1 gene expression of BAT was quantified after a single injection of some solution into interscapular. CL 316, 243 solution as a positive control responded, but there was no difference from control upon injection of CXCL 12 solution (Supplementary Fig. S4).

### Changes in metabolic parameters of CXCR4-deficient mice fed with HFD

**Figure 6:**
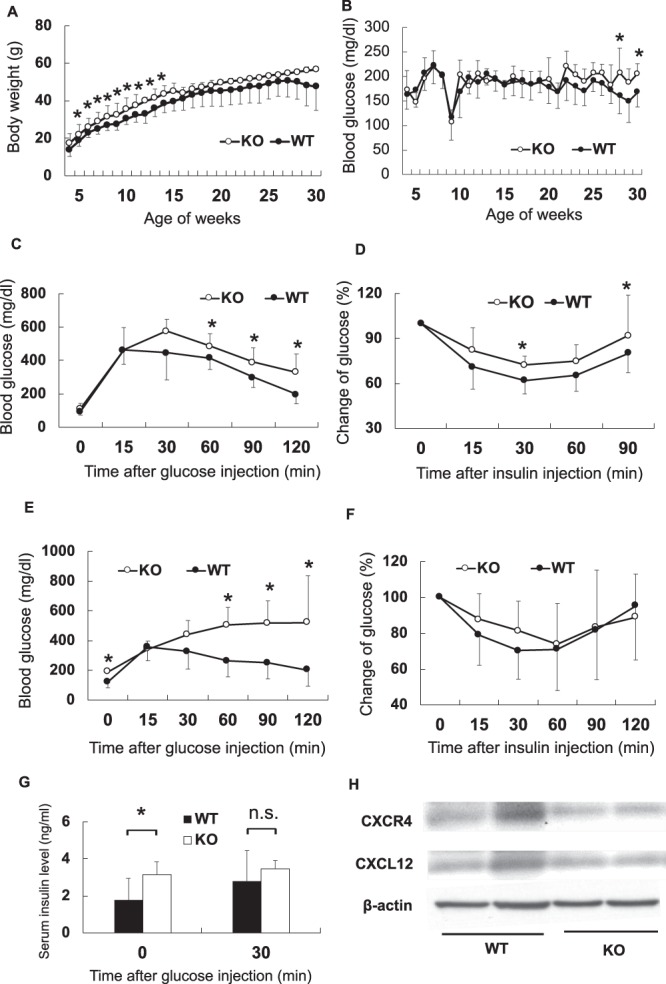
Metabolic factors in the knockout mice and wild type mice. Body weight and casual blood glucose were measured in the knockout mouse and sibling mouse every week after high-fat diet loading. Intra-peritoneal glucose tolerance test and Insulin tolerance test was demonstrated at 9 and 30 weeks old. The open and black circle represents the knockout mice and siblings. Black and open bar represent the knockout and the sibling mice, respectively. Values are shown as mean ± SD. All parameters are evaluated with unpaired Student’s *t*-test. *P < 0.05. n.s.: not a significant difference. WT: wild-type, CXCR4^flox/−^mice; KO: knockout, CXCR4^flox/flox^mice. (**A**) Change of body weight after high-fat diet load (n = 9–20). (**B**) Casual blood glucose (n = 4–16). Blood glucose in nine-week-old was lower than other time because it was measured at fasting condition to demonstrate glucose tolerance test. (**C**) Intra-peritoneal glucose tolerance test in nine weeks old (n = 4–16). (**D**) Insulin tolerance test in nine-week-old (n = 5–14). (**E**) Intra-peritoneal glucose tolerance test in 30 weeks old (n = 4–15). (**F**) Insulin tolerance test in 30 weeks old (n = 3–9). (**G**) Serum insulin concentration in 30 weeks old before and after glucose injection (n = 4–14). (**H**) CXCR4 and CXCL12 protein level in the BAT of the wild type and the knockout mice with the high-fat chow diet.

The change in the body weight was significantly higher for the knockout mice than for the control mice at the early phase of HFD feeding (Fig. 6A). The level of blood glucose increased in CXCR4-deficient mice at just 28 and 30 weeks of age as compared with the control mice (Fig. 6B). Glucose was injected into the intraperitoneal cavity to examine glucose metabolism after 4 weeks of HFD feeding. Blood glucose concentration increased in the knockout mice at 60, 90, and 120 min after glucose injection as compared with control mice (Fig. 6C). Insulin tolerance test (ITT) was performed to evaluate insulin sensitivity. Changes in glucose level in the knockout mice were significantly higher than those in the control mice at 30 and 90 min after insulin injection (Fig. 6D). Hence, these results suggest that glucose metabolism in CXCR4-deficient mice was deteriorated owing to insulin resistance.

We also evaluated glucose metabolism after 25 weeks of HFD feeding to investigate the effect of long-term exposure of HFD. Blood glucose levels were significantly higher in CXCR4-deficient mice than in the control mice at 0, 60, 90, and 120 min after glucose injection (Fig. 6E). No difference was observed in ITT results between two groups (Fig. 6F). Fasting serum insulin concentration increased in the knockout mice as compared with the control mice (Fig. 6G). However, no difference was observed in insulin concentration between two groups at 30 min after glucose injection. This result suggests that insulin sensitivity was deteriorated under fasting condition in CXCR4-deficient mice at 30 weeks of age.

### Other metabolic parameters in the BAT-specific CXCR4-dificient mice

Table 1 shows the nutritional and metabolic parameters of the mice fed with HFD at 9 and 30 weeks. CXCR4-deficient mice were heavier than the control mice. However, no difference was observed in the weight of BAT, WAT, and liver between both groups. Furthermore, no difference was reported in body temperature and lipid metabolism between two groups.

**Table 1:** Metabolic parameters at 9- and 30-week-old mice.

	WT (n = 18)	KO (n = 8)	P value
**9-week-old**			
Body weight (g)	28.37 ± 3.89	32.61 ± 5.89	0.030
BAT (g)	0.03 ± 0.013	0.024 ± 0.009	0.287
WAT (g)	1.09 ± 0.56	1.50 ± 0.40	0.239
Liver (g)	1.07 ± 0.23	0.94 ± 0.22	0.273
Body temperature (°C)	37.37 ± 0.37	37.06 ± 0.99	0.501
Triglyceride (mg/dL)	87.4 ± 26.8	75.2 ± 21.2	0.504
Total cholesterol (mg/dL)	148.8 ± 53.9	220.1 ± 54.7	0.109
LDL-C (mg/dL)	30.7 ± 23.2	64.3 ± 25.8	0.101
HDL-C (mg/dL)	107.7 ± 31.4	144.7 ± 29.0	0.134
**30-week-old**			
Body weight (g)	51.6 ± 14.9	58.8 ± 8.45	0.372
BAT (g)	0.097 ± 0.070	0.123 ± 0.032	0.494
WAT (g)	1.14 ± 0.11	1.47 ± 0.43	0.678
Liver (g)	2.54 ± 1.16	2.64 ± 1.02	0.897
Body temperature (°C)	36.5 ± 0.95	36.5 ± 0.57	0.997

To evaluate the inflammatory state of visceral fat, we assessed the level of TNF-a and IL-6 mRNA in visceral fat tissue of wild type and knockout mice. At the same time, Adiponectin was also evaluated in mice with normal chow and high-fat chow (Supplementary Fig. S3a–e). There was no significant difference.

To investigate the insulin resistance in the organ, the liver insulin signal was evaluated. There was no difference in phosphorylated Akt in the liver of knockout mouse with the high-fat chow (Supplementary Fig. S5).

To illustrate significantly changed biological process and pathways, we performed RNA-seq using BAT from wild type and the knockout mice. 119 genes were differentially expressing genes (DEGs) between wild type and knockout mice. STRING database showed that lipid metabolic process was the most significant biological process in these genes (Supplementary Fig. S61a). Dgat2 and Apoa family members were included in these groups (Supplementary Fig. S61b–d).

## Discussion

Results of the present study indicate that the CXCL12-CXCR4 pathway plays an essential role in the activation of the brown adipocytes through the P38 and ERK, but not PKA, signalling pathways. Body weight gain and glucose metabolism failure without CXCR4 in the BAT were observed along with insulin resistance after feeding mice with HFD.

The CXCL12-CXCR4 pathway seems to exert an entirely different effect on brown adipocytes or white adipocytes during glucose metabolism. We demonstrated the activating effect of the CXCL12-CXCR4 pathway solely on the brown adipocytes. Yao and colleagues reported that CXCL12 affected both white and brown adipocytes in CXCR4-deficient mice^15^. On the contrary, Shin reported that CXCL12 caused insulin resistance in white adipocytes^14^.

Obesity is associated with cardiovascular diseases, type 2 diabetes, and NASH^18^. The CXCL12-CXCR4 pathway is activated in these pathophysiologies, as previously reported and observed in the present study^11,13,14,19^. Obesity induces systemic elevation in CXCL12 concentration in the serum. Thermogenesis in the BAT results in the maintenance of homeostasis of glucose metabolism. However, the increased effects of this pathway on WAT than on BAT result in insulin resistance. Previous reports and the present study results suggest that this pathway contributes, at least in part, to insulin resistance associated with obesity.

Many activators of brown adipocytes have been reported, including noradrenaline^4^. In the present study, CXCL12 served as an activator of brown adipocytes. Brown adipocytes require various channel routes to maintain body temperature and metabolic homeostasis. Our results suggest that the CXCL12-CXCR4 pathway contributes to thermogenesis and maintains metabolic homeostasis in response to over-nutrition or intake of HFD.

CXCL 12 could activate BAT at high-fat chow diet loading. Even though CXCL 12 showed a high blood concentration with a high-fat diet, weight increased (Supplementary Fig. S7). The possibility is that the effect of suppressing body weight gain by the BAT activation is limited. Generally, short-term high-fat chow activates BAT, but weight increases^20,21^. The long-term high-fat chow lowers the function of BAT itself and further increases body weight^22^. Therefore, it seems that body weight gain was partially suppressed by BAT activation with CXCL 12 when BAT function is maintained at the initial stage of the high-fat chow (Fig. 6A).

The high-fat chow increased the concentration of serum CXCL 12 (Fig. 4C). Nobody knows where CXCL 12 was supplied during obesity. CXCL 12 is generally secreted from various cells and organs^23^. At the time of obesity, more CXCL 12 is secreted from the white adipocyte^14^. From our experiments, considering that the concentration of CXCL 12 systemically increased with high-fat chow, white adipocyte is most likely to be the main source of CXCL 12 in obesity. However, further studies are needed as it is the possibility that CXCL 12 is secreted more from other cells and organs during obesity.

The CXCL12-CXCR4 pathway facilitated thermogenesis via P38 and ERK pathways, but not the PKA pathway, in response to cold conditions^24,25^. Cold stimulation is fast and robust agitation in BAT activation to retain adequate body temperature avoiding the hypothermia. On the other hand, long-term exposure to HFD may serve as a continuous, gradual, and moderate stimuli for BAT activation to maintain homeostasis of nutritional metabolism.

The CXCL12-CXCR4 pathway and the adrenaline pathway in brown adipocyte activation are different.1; Receptors involved in the activation of brown adipocytes are CXCR4 for CXCL12 and β3 adrenergic receptors for adrenaline, so receptors are different. 2; PKA activation is essential for the adrenaline pathway in brown adipose activation^25^. In our experiments, PKA inhibitor (H 89) didn’t suppress activation marker *UCP-1* mRNA in CXCL 12 added cells (Fig. 3D). Therefore, downstream of CXCL 12 in brown adipocyte activation was not required PKA. From the consequences, we believe that both pathways are different in the brown adipocyte activation. Furthermore, considering the results of cold stimulation and high-fat chow loading on wild type mouse (Fig. 4A,B), adrenaline pathway works when acute brown adipocyte activation such as cold stimulation is required. When chronic brown adipocyte activation is required, CXCL12-CXCR4 may be necessary.

Activation of BAT was not observed by single injection of CXCL 12 (Supplementary Fig. S4). This result may indicate that short-term stimulation of CXCL 12 is not sufficient to activate BAT. There is no long-term CXCL 12 stimulation as a gain of function model in this study. Further studies will reveal the required stimulation time of CXCL 12 in BAT activation.

Dipeptidyl peptidase (DPP)-4 inhibitor is used for the treatment of type 2 diabetes without body weight gain. DPP-4 inhibitor activates the BAT through the inhibition of the degradation of incretin hormone that has the ability to lower glucose levels in humans and mice^26,27^. DPP-4 enzyme also quickly degrades CXCL12^28,29^. Our results suggest the CXCL12-CXCR4 pathway exerts a part of the effect of DPP-4 inhibitor on BAT activity and glucose level.

Some activators of BAT play a role of induction of the brown adipocyte or the beige cell such as interleukin 18^30–32^. However, the CXCL12-CXCR4 pathway did not facilitate *UCP-1* mRNA in white adipocytes (Supplementary Fig. S2). Expression of *PRDM16* as a surrogate marker of differentiation to brown adipocyte was stable in the CB-1 cells treated with CXCL12 (Fig. 2F). On the other hand, this pathway induces capillary endothelial cells in WAT^33^.

A limitation of our study is that the interaction between the CXCL12-CXCR4 pathway and other activators of brown adipocytes such as noradrenalin was not studied. The mechanism of insulin resistance in CXCR4-deficient mice was not precise in the present study. There was no difference in the insulin signal in the liver, however examining the insulin signal in other organs such as muscle and adipose tissue will reveal organs that cause insulin resistance in the knockout mouse. CXCR4 and CXL12 mRNAs in CB-1 cells were quantified, but protein levels were not evaluated. Quantification of protein expression reveals changes associated with adipocyte differentiation. Other of the limitations is that we didn’t demonstrate the experiment of mice under cold condition in the knockout mice. However, we speculated there was little difference between knockout and wild mice under cold condition because the level of CXCR4 mRNA in BAT already decreased in wild type mice under cold condition. The other limitation is not to evaluate the expression level of BAT CXCR4 protein in wild type mouse of a high-fat diet. Although CXCR4 mRNA was increased, the protein level may be decreasing conversely. Further research on this problem is necessary.

In conclusion, the present study demonstrates that the CXCL12-CXCR4 pathway activates brown adipocytes in obesity and is one of the diverse ways for BAT activation. This finding suggests the plausible role of this pathway in the development of promising treatment strategies for obesity and type 2 diabetes.

## Methods

### Ethics statement

All experimental procedures were performed in accordance with specified guidelines and regulations for the care and use of laboratory animals. All animal procedures were approved by the Chiba University Institutional Animal Care and Use Committee.

### Establishment of immortalised brown adipocytes

The BAT derived from C57BL/6 mice was finely chopped in balanced salt solution of Hanks with collagenase (C2139; Sigma) and bovine serum albumin (A9647; Sigma). Tissue samples were incubated with hot water at 37 °C for 30 min under constant shaking. The cell suspension was filtered through a 100-μm cell strainer and centrifuged at 500 × *g* for 5 min. The cells were cultured in Dulbecco’s modified Eagle’s medium (DMEM)- high glucose (D5796; Sigma) and immortalised by infection with SV40 lentivirus (Applied Biological Materials Inc.). Introduction of SV40 lentivirus into cells was confirmed from the expression of SV40 lentiviral gene with quantitative reverse transcription polymerase chain reaction (qRT-PCR) method. These cells were called as CB-1 cells.

### Differentiation of brown adipocytes

Confluent CB-1 cells were exposed to a differentiation medium. Differentiation medium is DMEM-high glucose containing 20 nM insulin (I6634; Sigma) and 1 nM T3 (T2877; Sigma). On the second day, the medium was changed to induction media, which comprised the differentiation medium plus 0.125 mM indomethacin (I7378; Sigma), 2 μg/mL of dexamethasone (D4902; Sigma), and 0.25 mM 3-isobutyl-1-methylxanthine (IBMX) (I7018; Sigma). On the third day, the medium was changed to differentiation medium. The differentiation medium was exchanged every 2 to 3 days after the fourth day. The state of CB-1 cells was observed, and the experiment was conducted in CB-1 cells with many lipid droplets in the cytoplasm after about 15 days from differentiation induction.

### Activation of brown adipocytes by CXCL12

Mature CB-1 cells were treated with 20 or 100 nM of recombinant CXCL12 (Murine SDF-1α: CXCL12 #250-20A; PEPROTECH) and incubated at 37 °C for 6 h. About 1 mM of CL316,243 (C5976; Sigma) as a selective β3AD-R agonist was added to mature CB-1 cells for the positive control of brown adipocyte activation.

### Evaluation of mRNA expression level

We extracted mRNA using RNeasy Mini Kit (QIAGEN). After treatment, CB-1 cells were washed thrice with phosphate-buffered saline and lysed by RLT buffer. The cell lysate was collected from the bottom of the culture dish using a scraper and transferred into the column of RNeasy kit. RNA was eluted using 20 μL of RNase-free water. Complementary DNA (cDNA) was prepared from the eluted RNA using QuantiTect Reverse Transcription Kit (QIAGEN). Expression of mRNA in the cells was examined by qRT-PCR (ABI7900; Applied Biosystems) with the cyber green method of the primer set (Supplementary Table S1). Level of mRNA were normalised to *36B4* gene.

### Detection of protein expression by western blotting

Proteins were extracted using the radioimmunoprecipitation assay (RIPA) buffer containing Complete (04693116001; Roche) as a protease inhibitor cocktail. The cell lysate was transferred into microtubes and centrifuged at 1,000 × *g* for 5 min. After collection of the supernatant, protein concentration was determined with the bicinchoninic acid method (Pierce® BSA Protein Assay Kit, Thermo Fisher Scientific). The samples were mixed with 6 μL of sodium dodecyl sulphate (SDS) sample buffer and the volume was adjusted to 20 μL using RIPA buffer. The samples were heated at 98 °C for 4 min and applied into the wells of 10% polyacrylamide gel® (ATTO) with electrophoresis apparatus. About 5 μL of Precision Plus Protein Standards (#161-0374; BIO-RAD) as protein markers were applied to a specific well of the polyacrylamide gel. The samples in the well were run at 0.04 A for 80 min. The separated protein bands were transferred onto polyvinylidene fluoride membranes (Sequi-Blot PVDF Membrane; BIO-RAD) at 20 V for 90 min. The membranes were blocked for 1 h at room temperature with Tris-buffered saline with 0.1% of Tween-20 (TBS-T) in 5% skim milk. After washing thrice with TBS-T, the membranes were incubated overnight at 4 °C on a shaker with anti-UCP-1 antibody (ab10983; Abcam) as the primary antibody. The membrane was washed with TBS-T and treated with an anti-rabbit IgG, horseradish peroxidase-linked Whole Antibody (NA934; GE Healthcare) in TBS-T as the secondary antibody for 1 h on a shaker at room temperature. Anti-β-actin antibody (A1978; SIGMA-ALDRICH) was used as an internal control. After washing thrice with TBS-T, the membrane was treated with enhanced chemiluminescence (ECL) mix (RPN2209; Amersham) for 5 min. The protein bands were exposed to an X-ray film in the dark and quantified using densitometry with ImageJ software (National Institute of Health).

### Measurement of oxygen consumption

Differentiated CB-1 cells were cultured in a 96-well plate and incubated at 37 °C for 40 min in a non-CO_2_ incubator. OCR and ECAR in each well were evaluated with XF 96 Extracellular Flux Analyzer (Seahorse Bioscience Inc.). Twelve measurements were approximately taken after every 7 min. CXCL12 and CL316,243 were added to each well via an injection device after the third measurement. The final concentrations of CXCL12 and CL316,243 were adjusted to 100 μM and 1 mM, respectively, in each well.

### Signal pathway experiment

Differentiated CB-1 cells were preincubated with P38, ERK, and PKA signal inhibitor at 37 °C for 30 min and treated with 100 nM of CXCL12 for 2 h. SB203580 (#5633; Cell Signalling Technology) as a P38 inhibitor, FR180204 (#0320; Sigma-Aldrich) as an ERK inhibitor, and H89 (#9844; Cell Signalling Technology) as a PKA inhibitor were adjusted to a final concentration of 10, 25, and 10 μM, respectively. At the end of the reaction, cell lysate was harvested using RLT buffer and RNeasy Mini kit. Extracted RNA was reverse transcribed to cDNA and examined with qRT-PCR to detect *UCP-1* mRNA expression.

### Phosphorylated p38 and phosphorylated ERK

CXCL 12 was added to CB-1 cells, and proteins were extracted using RIPA buffer after 15 minutes and 30 minutes. After western blotting, total p38(9212 S; Cell Signaling), phosphorylated p38(9211 S; Cell Signaling), total ERK (9102 S; Cell Signaling), and phosphorylated ERK (9106 S; Cell Signaling) were reacted as primary antibodies. After reacting with ECL using HRP secondary antibody, it was exposed to x-ray film, and the band was finally quantified.

### Mice with cold circumstances or HFD feeding

C57/B6 mice were placed at 4 °C as a cold environment for 4 h, and their BATs were excised after stimulation. C57/B6 mice as the control mice were left at 28 °C (room temperature) for 4 h. The expression of mRNA in the BAT was evaluated using qRT-PCR. An obesity model mouse induced with HFD was prepared. Four-week-old C57/B6 mice were fed with 60 kcal% of fat as a part of HFD (D12492; RESEARCH DIET INC.) for 4 weeks. Mice fed with 5 kcal% of fat as a normal fat diet (ND) (CE-2; CREA Japan Inc.) were regarded as a control group.

### Extraction of mRNA from tissues

The BAT was homogenised with 1 mL of TRIZOL reagent (15596018; Invitrogen) for ~30 mg of BAT. After homogenisation, the insoluble fraction was removed with centrifugation at 13,000 ×*g* for 5 min at 4 °C. The supernatant sample was treated with 0.2 mL of chloroform at room temperature for 5 min. The tube was vortexed for 15 s and centrifuged at 12,000 × *g* for 15 min at 4 °C. After centrifugation, the aqueous phase was transferred to a new tube and mixed with 0.5 mL of isopropyl alcohol. The sample was incubated at room temperature for 10 min and centrifuged at 12,000 × *g* for 10 min at 4 °C. The supernatant was removed, and the RNA pellet was washed once with 75% ethanol and centrifuged at 75,00 × *g* for 5 min at 4 °C. The RNA pellet was dried and 30 μL of RNase-free water was added to the pellet. The pellet was mixed by pipetting and incubated at 60 °C for 10 min. cDNA was prepared using QuantiTect Reverse Transcription Kit (205311; QIAGEN) from the obtained RNA, and mRNA expression was examined with qRT-PCR.

### Generation of knockout mice

CXCR4^flox^ mice were purchased from RIKEN BioResource Center (Ibaraki, Japan). UCP-1 Cre mice were provided by Charles River Labs Japan Inc. CXCR4^flox/flox^ mice were crossed with UCP-1 Cre mice to obtain F 2 generation of brown fat-specific CXCR4-deficient mice. Primary brown adipocytes derived from the BAT of these mice were cultured and the expression of mRNA was confirmed with qRT-PCR. BAT, WAT, and liver were excised from these mice, and the gene expression level was tested in each tissue. CXCR4^flox/−^ mice sibling was regarded as control mice.

### Exposure to HFD or normal chow diet

HFD containing 60 kcal% of fat was administered to male knockout mice and control mice until 32 weeks. Body weight and occasional blood glucose level were weekly measured. Food intake were estimated every five weeks.

### Evaluation of glucose metabolism

Intra-peritoneal glucose tolerance test (IPGTT) was carried out after 5 weeks of HFD or normal chow diet feeding. Mice were fasted for 15 h, and the glucose amount for injection was set at 2 g/kg. ITT was carried out to evaluate insulin sensitivity at 7 weeks after HFD or normal chow diet feeding. Mice were fed ad libidum and the amount of insulin was set at 0.6 units/kg. IPGTT was administered by the same protocol at 26 weeks after HFD or normal chow diet feeding. Insulin amount was set to 1.0 units/kg in ITT at 26 weeks. Blood was collected from the vein of the tail at fasting and 30 min after glucose loading. Serum insulin level was measured with Morinaga Ultra Sensitive Mouse/Rat Insulin ELISA Kit (M1104; Morinaga).

### Evaluation of CXCL12, and CXCR4 protein level in BAT

Proteins were extracted using RIPA buffer in the liver of wild type and the knockout mice. After western blotting, anti-CXCL12 (ab18919; abcam) antibody and anti-CXCR4 (ab124824; abcam) antibody were reacted as primary antibodies. After reacting with ECL using HRP secondary antibody, it was exposed to x-ray film, and the band was finally quantified.

### Measurement of serum CXCL12 concentration

The concentration of serum CXCL12 was measured at 5 and 26 weeks after feeding with HFD using R&D Systems Quantikine ELISA Mouse CXCL12/SDF-1α (#MCX120; R&D Systems).

### Single injection of CXCL12 in BAT of wild type mice

Wild type mice were injected phosphate buffered saline, CXCL12, or CL316, 243 as control, CXCL12, or positive control, respectively. Injection dose of CXCL12, and CL316,243 were 10 μg and 0.1μg base on 1 g body weight, respectively. Each BAT was excised 4 hours after injection and RNA was extracted. *UCP-1* mRNA levels as an activation marker for BAT were quantified by qRT-PCR.

### Quantification of Phosphorylated Akt in the Liver of Mice with High Fat Diet

Proteins were extracted from the liver by RIPA buffer. After electrophoresis of proteins, western blot is performed, and phosphorylated Akt antibody (2965 S; Cell Signaling) and total Akt (4691 S; Cell Signaling) antibody are reacted as a primary antibody, and ECL is added after reaction with a secondary antibody with HRP. It was taken in as an image after exposure to X-ray film.

### RNA-seq in BAT of the wild type and the knockout mice with the high-fat chow

A series of RNA-seq libraries was prepared in Kazusa DNA Research Institute.

We compared wild type and knockout mice with the R (Bio Conductor software package; version 3.4.1) and listed about 120 genes with P < 0.01. The biological process, KEGG pathway was obtained by analysing these genes into the String-database (https://string-db.org/). Furthermore, I made a figure of 12 genes included in the Positive regulation of lipid metabolism, which comes to the top by Biological process.

## Supplementary information

Supplementary information
