# Supplementary material for: CXCL12-CXCR4 pathway activates brown adipocytes and induces insulin resistance in CXCR4-deficient mice under high-fat diet
Source: Sci Rep. 2019 Apr 16;9:6165. doi: 10.1038/s41598-019-42127-8 (PMC6467900; doi:10.1038/s41598-019-42127-8)
Supplement: Supplementary file 1 — Supplementary information [file 41598_2019_42127_MOESM1_ESM.docx]

**Supplementary information**

**Full title: A CXCL12-CXCR4 pathway activates brown adipocytes and induces insulin resistance in mice without CXCR4 under high-fat diet load.**

**Authors: Kenichi Kurita^1,2^, Ko Ishikawa^1,2^, Kenji Takeda^1,2^, Masanori Fujimoto^1,2^, Hiraku Ono^1,2^, Jin Kumagai^1,2^, Hiromi Inoue^1,2^, Hidetaka Yokoh^1,2^, Koutaro Yokote^1,2^**

**Affiliations:** ^1^ Department of Diabetes Endocrinology, Hematology and Gerontology, Chiba University Graduate School of Medicine, 1-8-1 Inohana, Chuo-ku, Chiba 260-8670, Japan; ^2^Division of Metabolism and Endocrinology, Chiba University Hospital, 1-8-1 Inohana, Chuo-ku, Chiba 260-8670, Japan

***Corresponding:** Ko Ishikawa, MD, PhD,

Department of Endocrinology, Hematology and Gerontology, Chiba University Graduate School of Medicine, 1-8-1 Inohana, Chuo-ku, Chiba 260-8670, Japan

Division of Diabetes, Metabolism and Endocrinology, Chiba University Hospital, 1-8-1 Inohana, Chuo-ku, Chiba 260-8670, Japan

E-mail: ishikawako1225@gmail.com; Tel: +81-43-226-2089; Fax: +81-43-226-2095


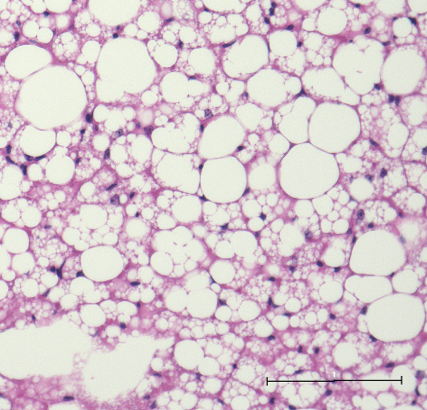

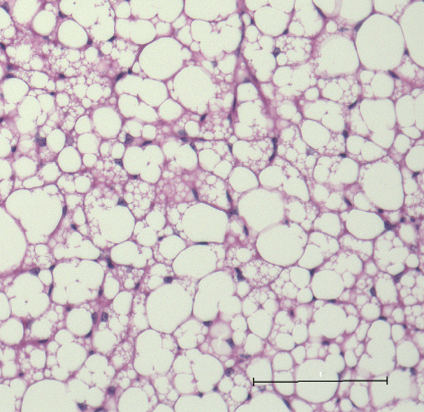


**A**

**B**

KO

Control

**D**

**C**


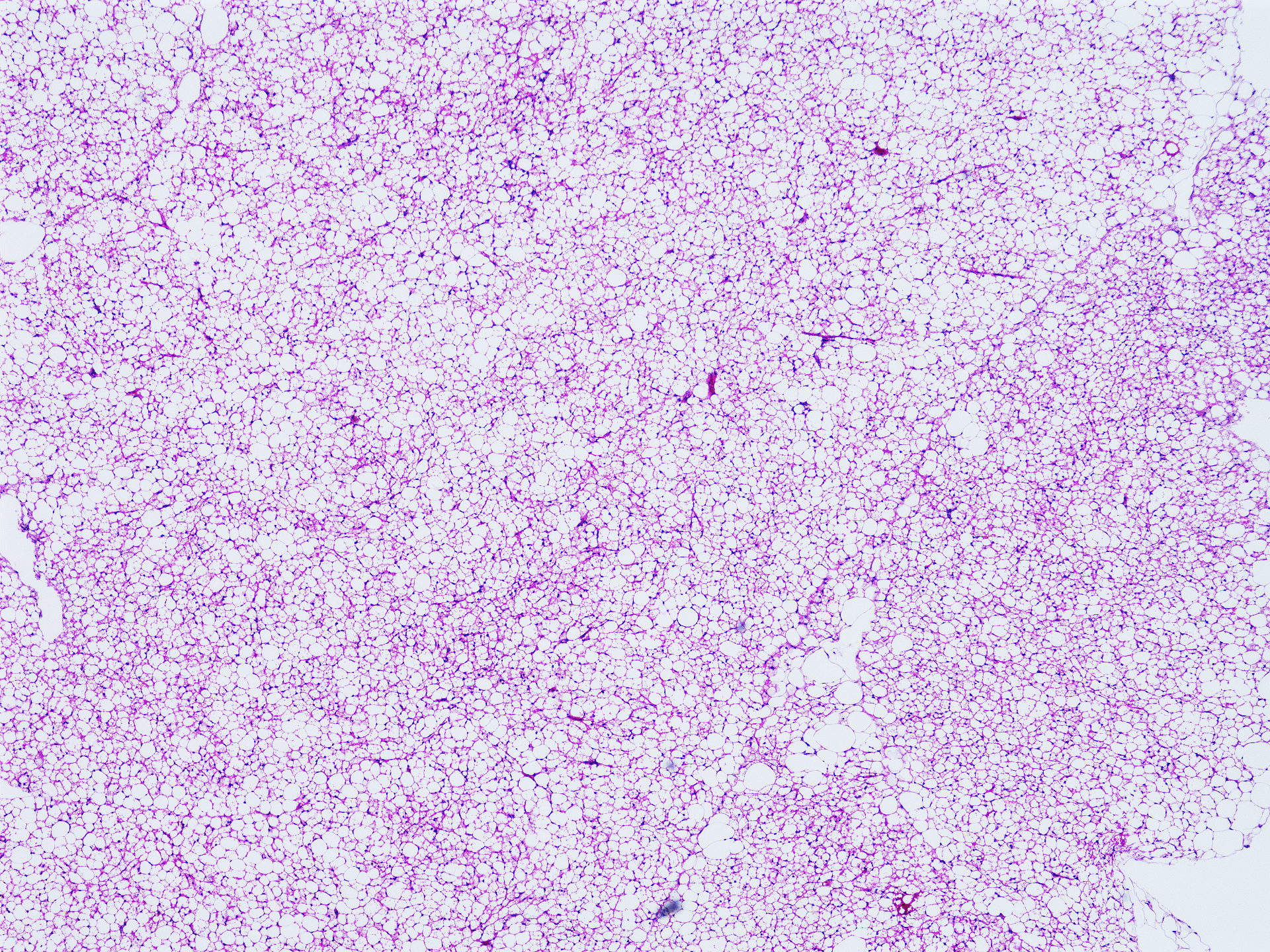

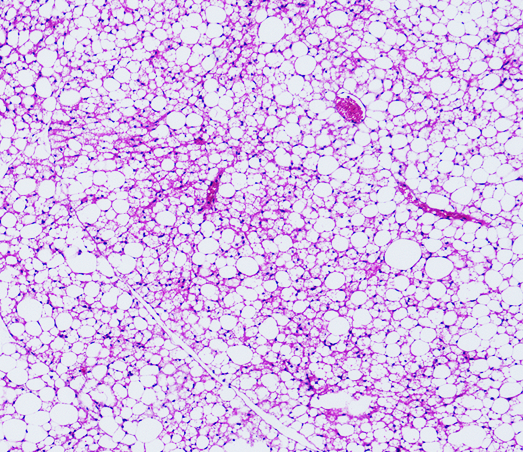


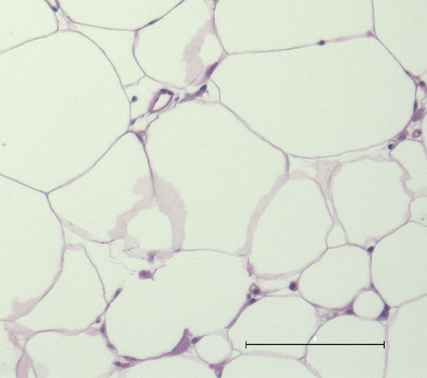


**E**

**F**

KO

Control

KO


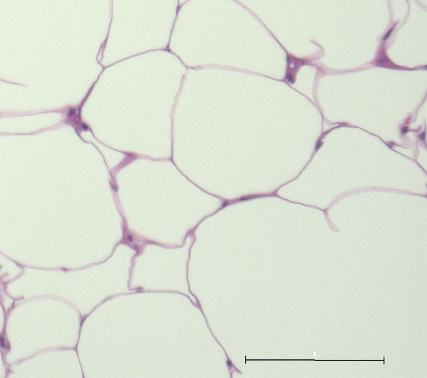


Control

**Supplementary Figure S1. Histological findings of the brown adipose tissue and the white adipose tissue in control and the knockout mice.**

(A, B, C, D) Histological examination in the brown adipose tissue of control and KO mouse in different scale. (E, F) Histological examination in the white adipose tissue of control and KO mouse. UCP-1 gene in BAT. Black scale is shown as 100 μm. WT: wild-type, CXCR4^flox/-^mice; KO: knockout, CXCR4^flox/flox^mice

UCP-1 mRNA levels

**Supplementary Figure S2. Expression of UCP-1 mRNA in white adipocytes treated with CXCL12 or CL316, 243.**

Primary white adipocyte was obtained from brown adipose tissue in C57/B6 mice and induced differentiation with differentiation media. We added 100 nM of CXCL12 or CL316, 243 to differentiated white adipocyte and evaluated UCP-1 mRNA using quantitative RT-PCR (n = 4). Black, open, and stripe rectangle are shown as control, CXCL12, and CL316, 243. Values are shown as mean ± SD.

**C**

**B**

**A**

**E**

**D**

**Supplementary Figure S3. Expression of adipokine mRNA in visceral fat in wild type and BAT-CXCR4 knockout mice.**

(A, B) TNF-a and IL-6 mRNA were genes association with inflammation. These mRNAs in visceral white adipose tissue of the mice loaded high fat diet were estimated by qRT-PCR. (C) Adiponectin, one of the adipokine, in visceral white adipose tissue of the mice with high fat diet was evaluated. (D, E) Adipokine in visceral white adipose tissue of the mice with normal chow. Values are shown as mean ± SD. WT: wild-type, CXCR4^flox/-^mice; KO: knockout, CXCR4^flox/flox^mice

*

UCP-1 mRNA levels

**Supplementary Figure S4. Injection of solution in interscapular to activate BAT of wild type mice.**

Wild type mice were injected phosphate buffered saline, CXCL12, or CL316, 243 as control, CXCL12, or positive control. Injection dose of CXCL12, and CL316,243 were 10 μg and 0.1μg base on 1 g body weight, respectively (n = 3 - 4). Values are shown as mean ± SD. Parameters are evaluated with Bonferroni's multiple comparison test. *P < 0.05.

**P-Akt**

**
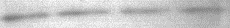

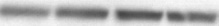
**

**WT**

**KO**

**Akt (PAN)**

**Supplementary Figure S5. Phosphorylation of Akt in the liver of the mouse with high-fat chow**

P-Akt: phosphorylated Akt; Akt (PAN): total Akt; WT: wild-type, CXCR4^flox/-^mice; KO: knockout, CXCR4^flox/flox^mice


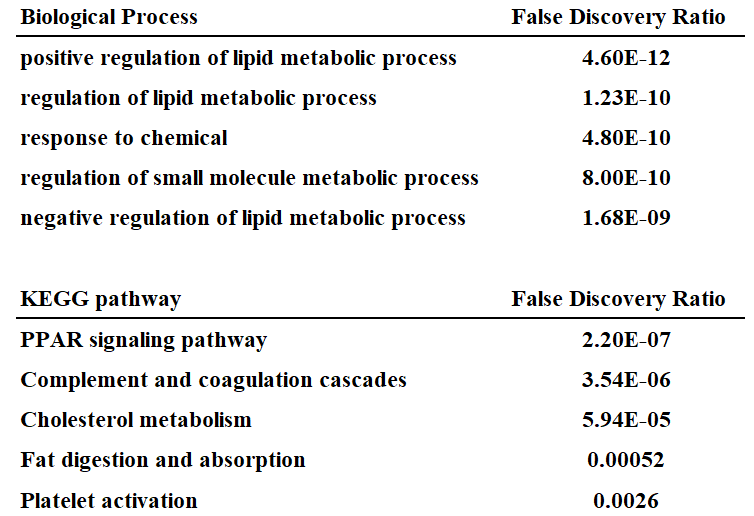


A


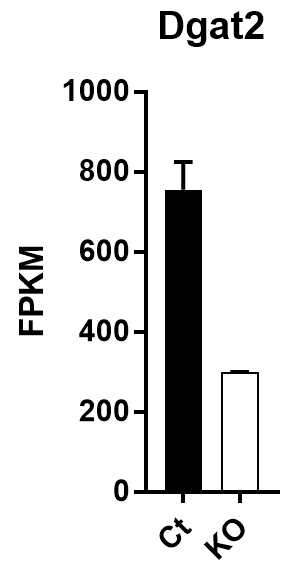

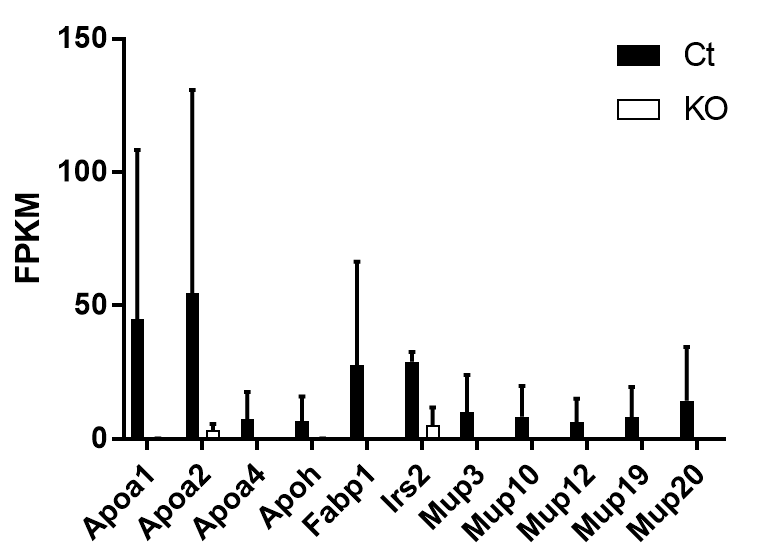


C

B


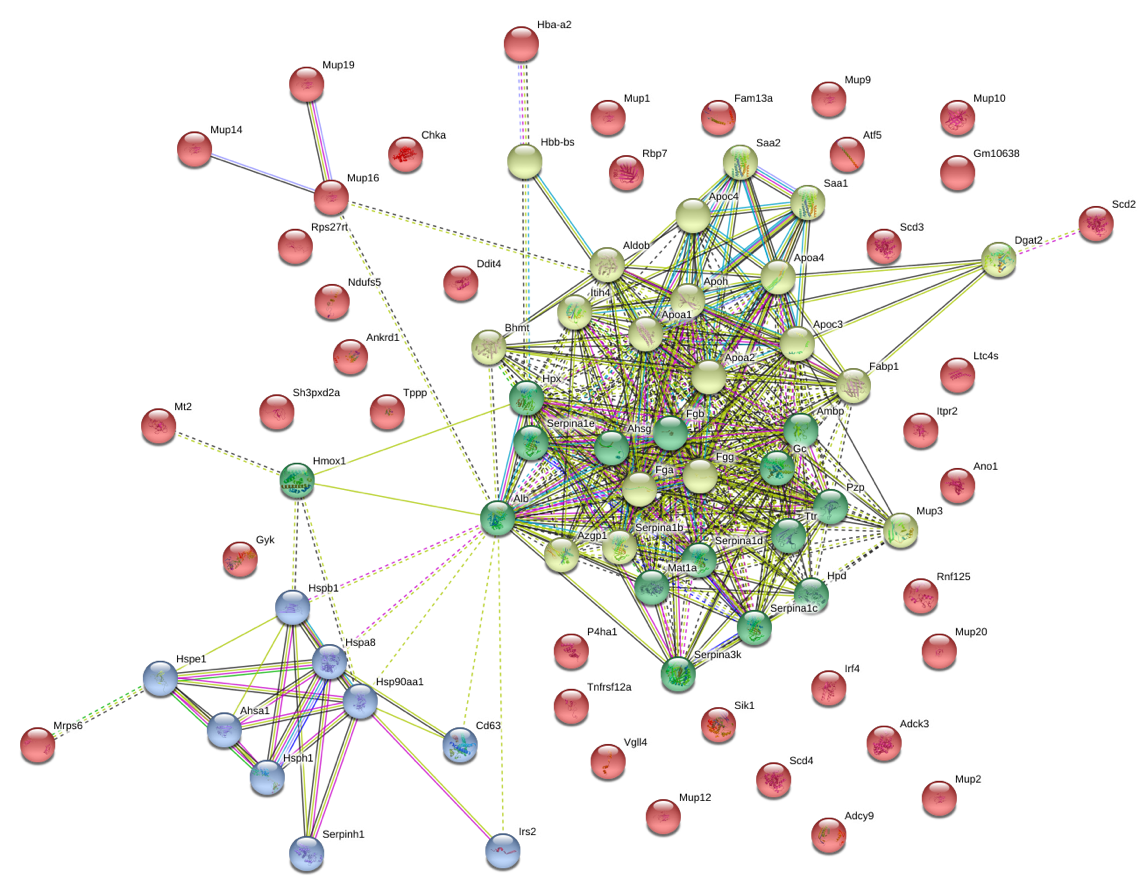


D

**Supplementary Figure S6. RNA sequence in the brown adipose tissue with high fat chow.**

(A) Significant biological process and KEGG pathways in 119 differentially expressing genes (DEGs) (p<0.01) between wild type and knockout mice. (B, C) Gene expression leveles of 12 genes included in the most significant biological process (positive regulation of lipid metabolic process). (D) Network analysis based on 119 DEGs. Ct: wild-type, CXCR4^flox/-^mice; KO: knockout, CXCR4^flox/flox^mice. DGAT2; Diglyceride acyltransferase 2, FPKM; Fragments Per Kilobase of exon per Million mapped fragments, KEGG; Kyoto Encyclopedia of Genes and Genomes.

**Supplementary Figure S7. Change of body weight in the wild type mouse with normal chow and the high-fat chow.**

ND: normal chow; HFD: high-fat chow

| Target Gene Name | Forward Primer | Reverse Primer |
| --- | --- | --- |
| UCP-1 | ACTGCCACACCTCCAGTCATT | CTTTGCCTCACTCAGGATTGG |
| CXCL12 | TGCATCAGTGACGGTAAACCA | TGTCTGTTGTTGTTCTTCAGCCGTGC |
| CXCR4 | TGCAGCAGGTAGCAGTGAAA | AAGTGTATACTGATCGGTTCCA |
| PGC-1α | CCCTGCCATTGTTAAGACC | TGCTGCTGTTCCTGTTTTC |
| m36B4 | AGATGCAGCAGATCCGCAT | GTTCTTGCCCATCAGCACC |
| Cidea | TGCTCTTCTGTATCGCCCAGT | GCCGTGTTAAGGAATCTGCTG |
| CytochromeC1 | GCTACCCATGGTCTCATCGT | CATCATCATTAGGGCCATCC |
| Glut4 | ACTCATTCTTGGACGGTTCCTC | CACCCCGAAGATGAGTGGG |
| CD36 | GCCAAGCTATTGCGACATGA | TCTCAATGTCCGAGACTTTTCAAC |
| β3-ADR | CCAGCCAGCCCTGTTGA | GGACGCGCACCTTCATAGC |
| PRDM16 | CAGCACGGTGAAGCCATTC | GCGTGCATCCGCTTGTG |
| TNF-α | TCTTCTCATTCCTGCTTGTGG | GGTCTGGGCCATAGAACTGA |
| Adiponectin | GGAGAGAAAGGAGATGCAGGT | CTTTCCTGCCAGGGGTTC |
| IL-6 | ACAAAGCCAGAGTCCTTCAGA | TGGTCCTTAGCCACTCCTTC |

**Supplementary Table S1. The list of primer set in this study.**

All quantitative RT-PCR was performed with SYBR Green method.
